# Population risk predictors of major adverse kidney events attributed to focal segmental glomerulosclerosis from the CURE-CKD registry

**DOI:** 10.1186/s12882-025-04334-6

**Published:** 2025-07-19

**Authors:** Susanne B. Nicholas, Lindsey M. Kornowske, Cami R. Jones, Kenn B. Daratha, Radica Z. Alicic, Christina L. Reynolds, Joshua J. Neumiller, Mark E. Bensink, Wu Gong, Keith C. Norris, Katherine R. Tuttle

**Affiliations:** 1 Department of Medicine, David Geffen School of Medicine at University of California, Los Angeles, 7-155 Factor Building, 10833 Le Conte Blvd, Los Angeles, CA 90095 USA; 2 Providence Medical Research Center, Providence Inland Northwest Health, Spokane, WA USA; 3 Department of Medicine, University of Washington, Seattle, WA USA; 4 College of Pharmacy, Washington State University, Spokane, WA USA; 5 Travere Therapeutics Inc., San Diego, CA USA; 6 Kidney Research Institute and Institute of Translational Health Sciences, University of Washington, Seattle, WA USA

**Keywords:** Focal segmental glomerulosclerosis, Population level predictors, Kidney function, Health insurance, Health care utilization, Clinical outcomes, Clinical decision-making

## Abstract

**Background:**

Predictors of major adverse kidney events (MAKE) in focal segmental glomerulosclerosis (FSGS) have not been previously explored within large, real-world populations. The study aim was to evaluate population-level predictors of MAKE attributed to FSGS from health system data.

**Methods:**

The study population was derived from electronic health records from Providence and University of California Los Angeles Health Systems. Identification of FSGS was based on International Classification of Diseases 9/10 diagnostic codes. Cox proportional hazards models were used to estimate the effects of traditional clinical and unique non-traditional variables including age, gender, race and ethnicity, health system, health insurance, healthcare utilization, estimated glomerular filtration rate (eGFR), diabetes, hypertension, and prescription medications as predictors of MAKE defined as: ≥ 40% eGFR decline, kidney failure (eGFR < 15 mL/min/1.73 m^2^, administrative codes for kidney failure, dialysis, or transplant) and death.

**Results:**

Adults with FSGS (N = 629) were 54% (n = 342) men and 53 ± 17 (mean ± SD) years old. Baseline eGFR was 60 ± 30 mL/min/1.73 m^2^, while median (interquartile range) urine albumin/creatinine ratio (UACR) and urine protein/creatinine ratio (UPCR) were 1,430 (520–2,630) mg/g and 1.6 (0.5–3.9) g/g, respectively. Angiotensin converting enzyme inhibitors or angiotensin receptor blockers were prescribed to 76% (n = 475), while corticosteroids and other immunomodulators were prescribed in 47% (n = 297) and 12% (n = 74), respectively. MAKE were observed in 42% (n = 262) of study participants over a median of 2.9 (1.4–4.5) years. Higher hazard for MAKE was associated with baseline above-median UACR or UPCR (HR [95% CI] (3.46 [2.28–5.23]) in patients with available measures, prescription for non-corticosteroid immunomodulator (1.87 [1.32–2.65]), non-commercial health insurance (1.78 [1.36–2.33]), hospitalization (1.64 [1.25–2.15]), lower eGFR per 10 mL/min/1.73 m^2^ 1.25 [1.18–1.32]), number of outpatient visits (1.03 [1.01–1.05]) and lower hazard for MAKE was associated with older age (0.89 [0.82–0.98]).

**Conclusions:**

Substantial loss of kidney function or kidney failure occurred in more than four in ten patients with FSGS by a median of three years. MAKE were predicted by unique population level factors, such as healthcare utilization and insurance type, which may help to identify patients with FSGS, who could most benefit from diagnostic testing and interventions to improve clinical outcomes.

**Clinical trial number:**

Not applicable.

**Supplementary Information:**

The online version contains supplementary material available at 10.1186/s12882-025-04334-6.

## Background

Focal segmental glomerulosclerosis (FSGS) is the most common cause of nephrotic syndrome and the most common primary glomerular disease in patients with kidney failure in the United States (US) [1]. FSGS describes a set of heterogeneous glomerular diseases in which some, but not all, glomeruli become sclerosed (focal), and involved glomeruli are only partially affected (segmental) [2, 3]. As FSGS progresses, the entire glomerulus becomes affected with more diffuse lesions and extensive accumulation of hyalinosis, podocyte effacement, and vascular obstruction [2, 4]. Although the exact incidence of FSGS is unknown, significant increases in kidney biopsy proven FSGS have ranged from 17% per five years between 1994 to 2003, to as high as 41% per five years between 2004 to 2013 [5]. More males than females are affected with FSGS [6].

FSGS may present with varying degrees of proteinuria and different rates of progression leading to major adverse kidney events (MAKE). Due to disease heterogeneity, identification of patient characteristics that predict progression and therapeutic response has been challenging. The mainstay of therapy for FSGS has traditionally included angiotensin converting enzyme (ACE) inhibitors or angiotensin receptor blockers (ARBs), glucocorticoids, and calcineurin inhibitors [7]. However, novel therapies have opened doors for various treatment options such that knowledge of key risk predictors would be instrumental in stratifying patients for timely interventions. Although several studies have previously examined characteristics associated with FSGS progression, these studies have been limited to small sample sizes and have reported varying results [8, 9, 10, 11, 12]. For instance, the presence of nephrotic syndrome predicts response to immunosuppression, and the histopathologic presence of interstitial inflammation was found to be a risk factor for kidney failure in a cohort of active-duty members of the US Department of Defense [13]. Nevertheless, specific predictors of MAKE have not been explored at a population level. The study aim was to evaluate traditional clinical and unique, non-traditional predictors of MAKE defined by ≥ 40% decline in estimated glomerular filtration rate (eGFR), kidney failure, and death in a large real-world population with FSGS.

## Methods

### Study population

**Fig. 1:**
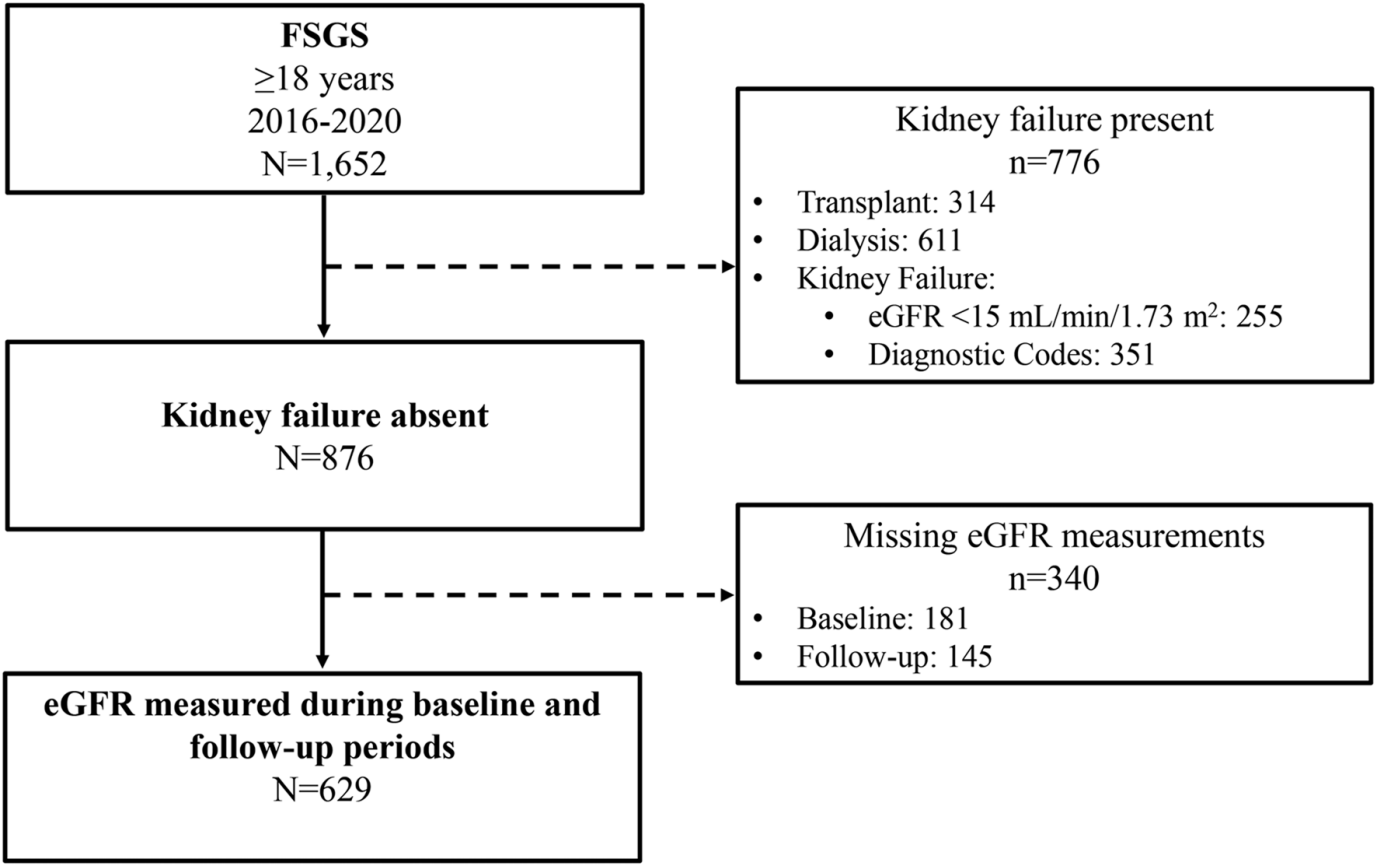
STROBE diagram for FSGS cohort selection. FSGS-focal segmental glomerulosclerosis, eGFR-estimated glomerular filtration rate

The Center for Kidney Disease Research, Education, and Hope (CURE-CKD) Registry [14, 15] contains demographics, physical and laboratory measures, prescription medications, and administrative codes from the Providence and UCLA Health Systems’ electronic health records (EHRs) for patients with chronic kidney disease (CKD) and at-risk for CKD by virtue of having diabetes, prediabetes, or hypertension. Identification of FSGS was based on International Classification of Diseases (ICD) 9/ICD 10 diagnostic codes. Baseline characteristics were determined from data for the 12-months prior to FSGS identification (Additional file 1) between January 1, 2016, and December 31, 2022. Patients were excluded for kidney failure or kidney replacement therapy defined by baseline eGFR < 15 mL/min/1.73 m^2^ or a diagnosis or procedure code indicative of kidney failure, transplant, or dialysis (Additional file 2). At least one eGFR measure (Chronic Kidney Disease Epidemiology 2021 equation) [16] during the baseline and follow-up periods were required for study inclusion (N = 629, Fig. 1). Diabetes and hypertension were identified as previously described for CURE-CKD [14]. All-cause death was ascertained from Providence EHR data using the Social Security Death Index [17], and from UCLA EHR data using the California death index [18]. Racial and ethnic identities were as reported in the EHR, and medication use was defined by an active prescription at baseline. Time-to-event analysis followed study participants from the index time until the first MAKE, or last encounter through December 31, 2022.

### Statistical analysis

Categorical variables are reported as frequencies and percentages. Continuous normally distributed variables are reported as mean ± standard deviation (SD), while continuous skewed variables are reported as median and interquartile range (IQR; Table 1). Median MAKE survival was estimated using the Kaplan-Meier method. Separate Kaplan-Meier estimates were constructed for the outcomes of all-cause death, ≥ 40% eGFR decline, kidney failure (eGFR < 15 mL/min/1.73 m^2^, administrative codes for kidney failure, dialysis, kidney transplant), and for composite MAKE grouped by eGFR category.

**Table 1:** Characteristics of patients identified with FSGS in 2016–2022

	Total	UACR/UPCR present	UACR/UPCR missing
**Demographics**			
Patients, n (% of total)	629 (100.0)	299 (47.5)	330 (5.2)
Gender, n (%)			
Women	287 (45.6)	134 (44.8)	153 (46.4)
Men	342 (54.4)	165 (55.2)	177 (53.6)
Race and ethnicity, n (%)			
American Indian or Alaska Native	10 (1.6)	3 (1.0)	7 (2.1)
Asian	83 (13.2)	45 (15.1)	38 (11.5)
Black	55 (8.7)	24 (8.0)	31 (9.4)
Hispanic or Latino(a)	28 (4.5)	12 (4.0)	16 (4.8)
Native Hawaiian or Pacific Islander	8 (1.3)	5 (1.7)	3 (0.9)
White	327 (52.0)	142 (47.5)	185 (56.1)
Other^1^ or missing	118 (18.8)	68 (22.7)	50 (15.2)
Age, y, mean, SD	53, 17	53, 18	52, 17
Age Category			
18-39	183 (29.1)	86 (28.8)	97 (29.4)
40-59	206 (32.8)	93 (31.1)	113 (34.2)
60-79	214 (34.0)	106 (35.5)	108 (32.7)
≥ 80	26 (4.1)	14 (4.7)	12 (3.6)
Primary health insurance, n (%)			
Commercial	371 (59.0)	188 (62.9)	183 (55.5)
Medicaid	75 (11.9)	28 (9.4)	47 (14.2)
Medicare	169 (26.9)	81 (27.1)	88 (26.7)
Missing/Unknown	14 (2.2)	2 (0.7)	12 (3.6)
**Health System Characteristics and** ** Health Care Utilization**			
Health System, n (%)			
UCLA Health	204 (32.4)	111 (37.1)	93 (28.2)
Providence	425 (67.6)	188 (62.9)	237 (71.8)
Outpatient Visits, median (IQR)	18 (8–33)	23 (12–38)	12 (6–29)
Hospitalization, n (%)	181 (28.8)	73 (24.4)	108 (32.7)
**Baseline Medication Coverage (≥45 d), n (%)**			
Medication use, n (%)			
ACE inhibitor/ARB	475 (75.5)	237 (79.3)	238 (72.1)
Corticosteroid	297 (47.2)	139 (46.5)	158 (47.9)
Other immunomodulator^2^	74 (11.8)	29 (9.7)	45 (13.6)
SGLT2 inhibitor	22 (3.5)	15 (5.0)	7 (2.1)
**Baseline Clinical Characteristics**			
Hypertension, n (%)	535 (85.1)	264 (88.3)	271 (82.1)
Diabetes, n (%)	245 (39.0)	140 (46.8)	105 (31.8)
eGFR, mL/min/1.73 m^2^, n (%)	629 (100.0)	299 (100.0)	330 (100.0)
mean, SD	60, 30	60, 30	60, 30
eGFR category, n (%)			
≥ 90	128 (20.3)	60 (20.1)	68 (20.6)
60-89	153 (24.3)	75 (25.1)	78 (23.6)
45-59	104 (16.5)	44 (14.7)	60 (18.2)
30-44	140 (22.3)	69 (23.1)	71 (21.5)
15-29	104 (16.5)	51 (17.1)	53 (16.1)
UACR, mg/g, n (%)	233 (37.0)	233 (77.9)	-
median (IQR)	1430 (520–2630)	1430 (520–2630)	-
UPCR, g/g, n (%)	119 (18.9)	119 (39.8)	-
median (IQR)	1.6 (0.5–3.9)	1.6 (0.5–3.9)	-
Systolic blood pressure, mmHg, n (%)	592 (94.1)	289 (96.7)	303 (91.8)
mean, SD	132, 16	133, 16	132, 17
BMI, n (%)	580 (92.2)	281 (94.0)	299 (90.6)
mean, SD	30, 7	30, 8	30, 7
HbA1c, %, n (% of diabetes)	159 (64.9)	100 (71.4)	59 (56.2)
mean, SD	7.0, 1.6	7.1, 1.8	6.9, 1.4

FSGS-Focal Segmental Glomerulosclerosis, UACR-urine albumin/creatinine ratio, UPCR-urine protein/creatinine ratio, SD-standard deviation, UCLA-University of California, Los Angeles, IQR-interquartile range, ACE-angiotensin converting enzyme, ARB-angiotensin II receptor blocker, SGLT-sodium-glucose co-transporter, eGFR-estimated glomerular filtration rate; BMI-body mass index, Hb-hemoglobin

^1^includes patients that did not identify with main census categories

^2^includes biologics, calcineurin inhibitors, cytotoxic agents, mTOR inhibitors, and pyrimidine synthesis inhibitors

Cox proportional hazards models were used to estimate the effects of clinical and non-clinical variables on the hazard of MAKE in patients with FSGS. Unadjusted and adjusted models were constructed from a set of baseline clinical variables including eGFR (per − 10 mL/min/1.73 m^2^), diabetes and hypertension status (yes/no), and prescription medications including ACE inhibitors, ARBs, corticosteroids, and other immunomodulators (yes/no; including biologics, calcineurin inhibitors, cytotoxic agents, mammalian target of rapamycin inhibitors, corticotropin agents, and pyrimidine synthesis inhibitors). Demographic variables included age (per 10 years), gender (women or men), and race and ethnicity (non-White versus White, non-Hispanic). Health care variables were health system (Providence versus UCLA Health), primary payer for health insurance (non-commercial versus commercial), hospitalization (yes/no) and frequency of outpatient visits (number) in the 12-month baseline period. Exploratory subgroup analyses included 1) a fully adjusted Cox model including status above versus below the cohort median urine albumin/creatinine ratio (UACR; 1,430 mg/g) or urine protein/creatinine ratio (UPCR; 1.6 g/g) for the subset of participants with baseline period UACR or UPCR measurements (N = 299) and 2) unadjusted Cox models to examine association of variables with MAKE by eGFR category. Alpha was selected as < 0.05 *a priori* to define statistical significance. A Bonferroni correction adjusted for multiple comparisons used to test an interaction term for each variable and eGFR category (P < 0.001) in the exploratory subgroup analysis. Analyses were performed in R version 4.2.2 [19] with the Survival package version 3.5–5 [20] and Prodlim package version 2023.03.3 [21].

## Results

### Baseline characteristics

The FSGS cohort (N = 629) was 54% men (n = 342/629) and 53 ± 17 years of age (Fig. 1, Table 1). Racial identity was Black in 8.7% (n = 55/629), Asian in 13.2% (n = 83/629) and White in 52% (n = 327/629). Ethnic identity was Hispanic or Latino(a) in 4.5% (n = 28/629). The proportion of participants with commercial health insurance as the primary payer was 59% (n = 371/629), while 27% (n = 169/629) of patients had Medicare and 12% (n = 75) had Medicaid [22]. Hypertension and diabetes were present in 85% (n = 535/629) and 39% (n = 245/629), respectively. Baseline eGFR (mean ± SD) was 60 ± 30 mL/min/1.73 m^2^ and the median (IQR) UACR and UPCR were 1,430 (520–2,630) mg/g and 1.6 (0.5–3.9) g/g, respectively. Prescription medications included ACE inhibitors/ARBs (76%, n = 475/629), corticosteroids (47%, n = 297/629), and other immunomodulators (12%, n = 74/629; Table 1). The characteristics of patients identified with FSGS according to eGFR category at baseline for model variables are shown in Additional file 3.

### Rates and predictors of major adverse kidney events

**Fig. 2:**
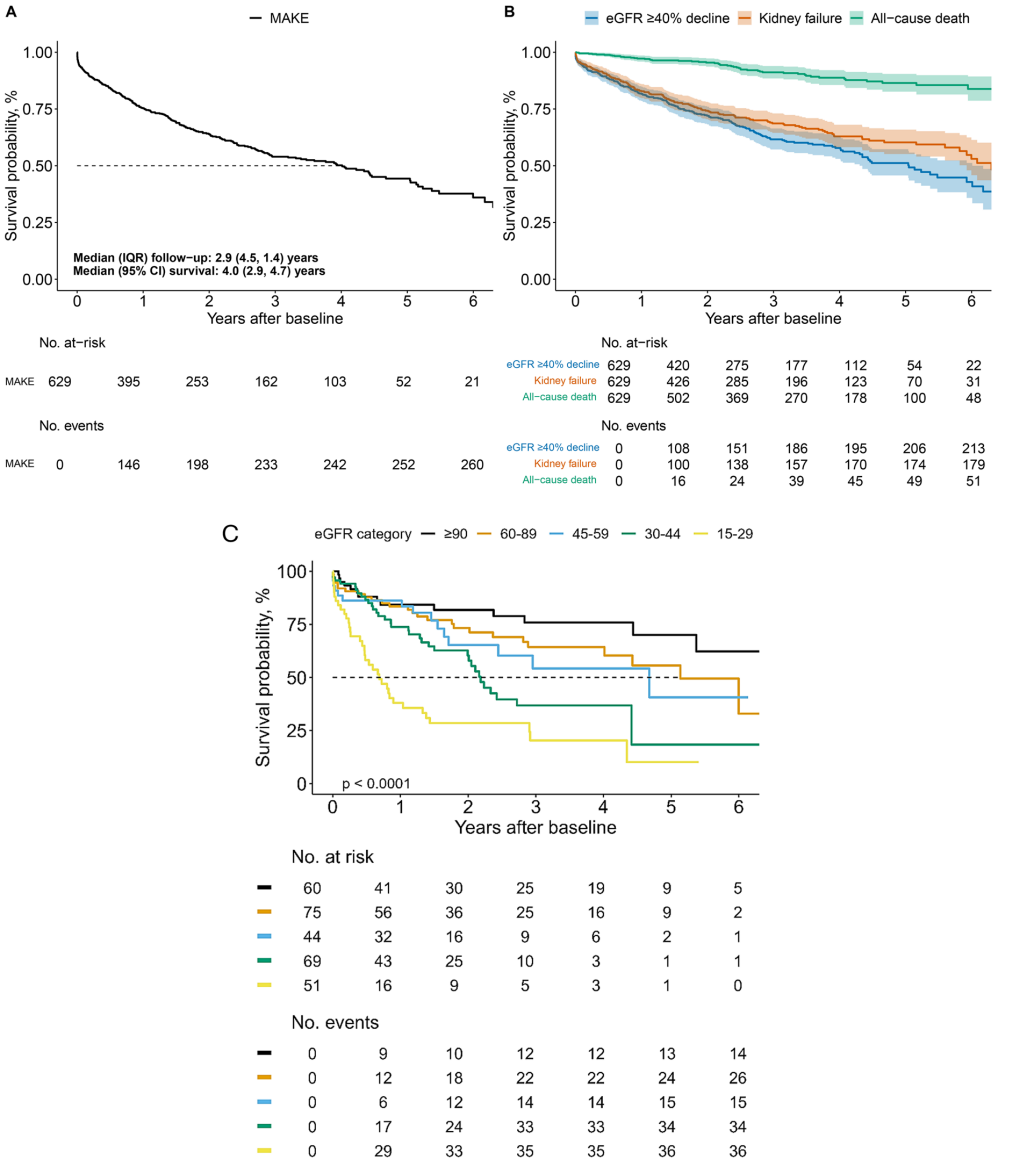
Survival probability of MAKE in patients with FSGS Kaplan-Meier survival curves. **A.** Primary composite MAKE outcome ≥ 40% eGFR decline, eGFR < 15 mL/min/1.73 m^2^, dialysis or transplant, and death in patients with FSGS (N = 629). **B.** Individual components for the primary composite MAKE outcome. **C**. Subgroup analysis according to eGFR category. MAKE-major adverse kidney events, eGFR-estimated glomerular filtration rate

MAKE were observed for 42% (n/N = 262/629) of study participants over a median (IQR) follow-up of 2.9 (1.4–4.5) years (Fig. 2A). Median (95% confidence interval [CI]) survival without MAKE was 4.0 (2.9–5.0) years. First MAKE was 51% (133/262) for ≥ 40% eGFR decline, 28% (74/262) for eGFR < 15 mL/min/1.73 m^2^, 11% (28/262) for dialysis, 5% (13/262) for kidney transplant, and 5% (14/262) for all-cause death (Table 2). The frequency of individual MAKE components by eGFR category showed that 40% eGFR decline events were more common among groups with higher eGFR categories (≥90-45 mL/min/1.73 m^2^), and that kidney failure events, dialysis and all-cause death were proportionately greater among those in the lower eGFR categories (30–44 and 15–29 mL/min/1.73 m^2^; Additional file 4).

**Table 2:** Summary of outcomes among study participants with FSGS and MAKE over median follow-up of 2.9 years (2016–2022)

	Total	Measured for UACR/UPCR	Not Measured for UACR/UPCR
Total with MAKE, n (% of cohort)	262 (41.7)	125 (41.8)	137 (41.5)
**Event type, n (% of N with MAKE)**			
eGFR ≥ 40% decline	133 (50.8)	62 (49.6)	71 (51.8)
Kidney failure or kidney replacement therapy			
eGFR < 15 mL/min/1.73 m^2^	74 (28.2)	33 (26.4)	41 (29.9)
Dialysis	28 (10.7)	17 (13.6)	11 (8.0)
Transplant	13 (5.0)	6 (4.8)	7 (5.1)
All-cause death	14 (5.3)	7 (5.6)	7 (5.1)

MAKE-major adverse kidney events, FSGS-focal segmental glomerulosclerosis, UACR-urine albumin/creatinine ratio, UPCR-urine protein/creatinine ratio, eGFR-estimated glomerular filtration rate

Kaplan-Meier estimates for deaths (8%; n/N = 52/629), kidney failure (29%; n/N = 181/629), and ≥ 40% eGFR decline (34%; n/N = 215/629) as independent outcomes are shown in Fig. 2B. The results of Kaplan-Meier estimates of MAKE survival by eGFR category indicate that groups with lower eGFR categories (30–44 and 15–29 mL/min/1.73 m^2^) tended to have lower survival compared to those with higher eGFR (≥90, 60–89 and 45–59 mL/min/1.73 m^2^; Fig. 2C.

**Fig. 3:**
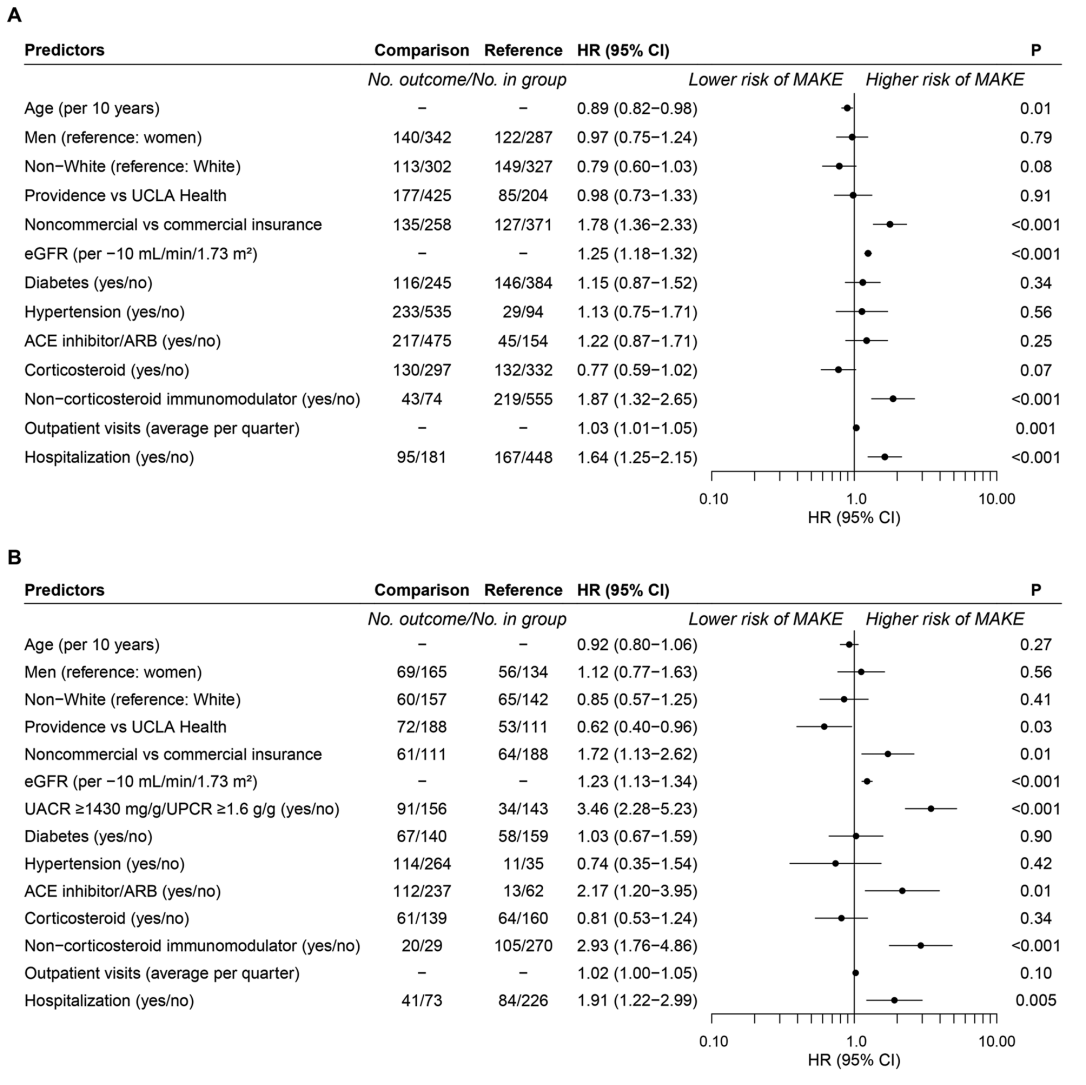
Predictors of MAKE in patients with FSGS. Adjusted Cox proportional hazards models **A.** Main model with full cohort. **B.** Secondary model including subset with baseline UACR or UPCR measurements. MAKE-major adverse kidney events, HR-hazard ratio, CI-confidence interval, UCLA-University of California Los Angeles, eGFR-estimated glomerular filtration rate, ACE-angiotensin converting enzyme, ARB-angiotensin II receptor blocker, UACR-urine albumin/creatinine ratio, UPCR-urine protein/creatinine ratio (in A and B use non-corticosteroid immunomodulator)

In the main analysis, baseline prescription for non-commercial health insurance (HR = 1.78, 95% CI = 1.36–2.33), lower baseline eGFR per 10 mL/min/1.73 m^2^ (HR = 1.25, 95% CI = 1.18–1.32), non-corticosteroid immunomodulators (hazard ratio [HR] = 1.87, 95% CI = 1.32–2.65), more frequent outpatient visits (HR = 1.03, 95% CI = 1.01–1.05) and hospitalization (HR = 1.64, 95% CI = 1.25–2.15) predicted greater hazard for MAKE (Fig. 3A). Notably, older age was associated with lower hazard for MAKE (HR = 0.89, 95% CI = 0.82–0.98).

In the exploratory analysis of the subgroup with UACR or UPCR measurements during the baseline period, similar overall relationships as with the main analysis were observed (Additional file 2). UACR ≥ 1,430 mg/g or UPCR ≥ 1.6 g/g predicted higher MAKE hazard (HR = 3.46, 95% CI = 2.28–5.23; Fig. 3B). Other predictors were prescriptions for non-corticosteroid immunomodulators (HR = 2.93, 95% CI = 1.76–4.86) and ACE inhibitors or ARBs (HR = 2.17, 95% CI = 1.20–3.95), hospitalization (HR = 1.91, 95% CI = 1.22–2.99), non-commercial health insurance (HR = 1.72, 95% CI = 1.13–2.62), and lower eGFR per 10 mL/min/1.73 m^2^ (HR = 1.23, 95% CI = 1.13–1.34). Differing from the main analysis, patients from Providence compared with UCLA Health had lower hazard for MAKE (HR = 0.62, 95% CI = 0.40–0.96).

The exploratory analysis of unadjusted Cox proportional hazards models by eGFR category showed that variable associations with MAKE were generally consistent with the main analysis (Additional file 5). There was no evidence for interaction by eGFR category after correction for multiple comparisons.

## Discussion

Our study assessed clinical and unique non-traditional population-level predictors of MAKE, not previously recognized to predict clinical outcomes or facilitate risk stratification in patients with FSGS. We showed that MAKE were highly common among a diverse cohort of adult patients with FSGS in contemporary clinical practice settings at two large health systems in the US. MAKE occurred in 42% of patients with FSGS and a mean baseline eGFR of 60 mL/min/1.73 m^2^ over a relatively short median follow-up of 2.9 years. Of patients who experienced MAKE, 51% had a ≥ 40% decline in eGFR, while nearly as many (44%) had kidney failure defined by eGFR < 15 mL/min/1.73 m^2^, dialysis, or transplant as a first event. Higher hazard for MAKE was predicted by reduced kidney function, and non-commercial health insurance at the time of FSGS identification, as well as prescriptions for non-corticosteroid immunomodulators, hospitalizations, and more frequent outpatient visits. On the other hand, older age was associated with lower hazard for MAKE, implying that younger age might be an important factor in more aggressive forms of FSGS presentation, as was previously demonstrated [23], and that these individuals should likely be considered for early diagnostic biopsy to guide therapeutic intervention.

The subgroup analyses by eGFR category provided important insights. For example, hypertension was common across all levels of kidney function, and patients with reduced kidney function were more likely to have non-commercial insurance and to be prescribed ACE inhibitors/ARBs. Importantly, there were trends for lower survival in groups with lower eGFR, and despite few observed events, findings of risk predictors for MAKE by eGFR category were generally consistent with the main analysis.

In an exploratory analysis of the subset of patients with baseline albuminuria or proteinuria measures, having above-median UACR/UPCR (≥1,430 mg/g UACR or ≥ 1.6 g/g UPCR) predicted a more than three-fold greater hazard for MAKE compared to study participants with measures below the median, with non-commercial health insurance, reduced kidney function, prescription for ACE inhibitors/ARBs and non-corticosteroid immunomodulators, and hospitalizations, suggesting these patients are sicker and had consistent risks for MAKE. Interestingly, a cohort of US Veteran patients with FSGS and government-funded health insurance had significant healthcare resource utilization, with ~ 40% having inpatient admission and 33% having emergency department visits within the first year following their FSGS diagnosis [24]. However, patients with access to only non-commercial insurance may face added limitations that could further negatively impact their disease diagnosis and treatment, possibly due to poor access to healthcare services, especially visits to specialists. Our study is the first to our knowledge to show that FSGS patients with non-commercial health insurance also have increased risk for MAKE, implying that these patients may need enhanced care. Notably, the increased risk of MAKE observed with prescriptions for ACE inhibitors/ARBs, which are cornerstone therapies to slow proteinuric CKD progression, may potentially be attributed to association with acute decreases in eGFR among hospitalized patients with multiple co-morbidities, hyperkalemia, medication non-adherence [25–28] or confounding by indication. Interestingly, in the albuminuria or proteinuria subset, health system emerged as a predictor of MAKE with lower risk within Providence versus UCLA Health. This a relevant issue as health system utilization has been shown to impact clinical outcomes for patients with nephrotic syndrome. A study of 1,232 patients with nephrotic syndrome showed that patients who were denied access to prescribed immunosuppressive therapy also had higher inpatient admissions [29]. However, in the main analysis of the current study, there was no difference in MAKE among patients receiving care at either Providence or UCLA Health, which suggests possible differences in populations or care processes, e.g. albuminuria or proteinuria testing, between systems may exist.

Historically, a number of factors have been recognized as predictors of CKD progression in patients with FSGS, but proteinuria remains a hallmark prognostic indicator of poor kidney survival, with > 50% of patients progressing to kidney failure [4, 11, 30, 31]. In FSGS, nephrotic range proteinuria (>3.5 g/24 h) typically represents significant podocyte effacement and podocyte loss, particularly if podocyte depletion is greater than 40%, in which case this picture is characteristically associated with reduced kidney function [32]. The results of our study are consistent with the known impact of elevated proteinuria, even at levels lower than nephrotic range as a significant predictor of MAKE. In addition to proteinuria, histopathologic lesions have been implicated as highly predictive of poor clinical outcomes in FSGS [33]. In the Nephrotic Syndrome Study Network, a cohort of 224 participants with FSGS who were biopsied between 2010 and 2017, histologic features that were most predictive of poor clinical outcomes in patients included not only the typical lesions of FSGS, but also lesions of interstitial fibrosis and tubular atrophy, among other characteristics such as adhesion, periglomerular fibrosis, and acute tubular injury [33]. While certain histologic variants have important prognostic value that may distinguish primary from secondary forms of FSGS [34, 35, 36], no histopathologic lesion is pathognomonic of primary FSGS [3], and as such other clinical features may be more relevant when assessing and predicting risk for clinical outcomes.

Several studies have tested the association of clinical features with various outcomes in FSGS. Age ≥ 60 years and eGFR < 60 mL/min/1.73 m^2^ have been uncovered as independent risks for reaching a 50% decline in eGFR or kidney failure [37]. Higher baseline serum creatinine has also been found to be a positive predictor of kidney failure even when both clinical and histological parameters are evaluated [38, 39]. Similar to our study, however, body mass index and hypertension have not proven to have prognostic value in FSGS [4, 11, 36].

MAKE is now an accepted composite kidney disease outcome and has been routinely used to facilitate comparisons of patient-centered, primary and secondary outcomes across clinical trials [40–42]. Additionally, predictors of MAKE may permit selection of patients who could most benefit from early kidney biopsy to guide clinical decision making. Indeed, predictive models have successfully incorporated combinations of clinical, biochemical, and pathological features to evaluate the risk for CKD progression, kidney failure, and all-cause mortality in FSGS patients [35, 39, 43–47]. However, the inclusion of other indicators such as medication (e.g., ACE inhibitors/ARBs, non-corticosteroid immunomodulators) and health care utilization (e.g., frequency of outpatient visits and hospitalization) may also be useful in risk stratification for more appropriate and timely management of FSGS. With the advent of new and improved therapies for FSGS, predicting MAKE for risk stratification can help facilitate precision care. As such, the results of our study may have important implications for clinical practice indicating that in addition to known clinical factors, there are non-clinical predictors that should be included in algorithms to identify high-risk patients who may benefit from close monitoring and intervention.

### Strengths and limitations

There are several strengths to the current study. The dataset consists of extensively curated data from two large, geographically distinct health systems consisting of demographics, laboratory measurements, active prescription records, and unique parameters of insurance status and records of inpatient and outpatient visits. Of note, CURE-CKD is one of the largest registry cohorts comprised of over 600 patients with FSGS treated in real-world clinical practice. Conversely, the use of EHR data presents limitations including the use of diagnostic codes to establish diagnosis of FSGS, missing data, analysis of retrospective data, potential miscoding of conditions in the absence of a kidney biopsy diagnosis, inability to verify medication taking, race and ethnicity information that may not correctly reflect self-reported race and ethnicity and recording of health insurance status that may change over time. Importantly, a contemporary diagnosis of FSGS may be designated by clinical parameters without kidney biopsy, therefore some clinical diagnoses of FSGS may not have been confirmed by histopathology. This is considered an important limitation in the current study. As the study was primarily population-based, it was not intended to (re)define the biology of FSGS, as in studies that examined specific histologic predictors in modeling FSGS outcome, or studies that required a biopsy-proven diagnosis to assess response to therapy [48–51], and thus the lack of biopsy data may have impacted the overall study findings. For example, patients with FSGS may have been misclassified, and this may have affected patient selection for immunomodulator therapy. Further, the absence of histopathology, such as from detailed medical records reviews, limited opportunities to identify potential secondary causes and sub-types of FSGS. Indeed, inability to differentiate secondary causes (e.g. due to viral infections or drug toxicities) and specific FSGS subtypes (e.g. collapsing, cellular variants) [52–54] obscured pathological heterogeneity precluding assessment of MAKE, predictors in the specific subgroups of FSGS. Even so, the absence of FSGS sub-types did not alter the startling finding that 42% of patients identified by ICD 9/10 codes in a real-world health care setting experienced MAKE in a median follow-up of 2.9 years. Indeed, these rates may not be representative of rates seen in other health systems, depending on differences in the unmeasured classification of FSGS sub-type. Finally, the study results did not examine post-transplant FSGS, or the effect of responsiveness to treatment and were limited to two, albeit large health systems, and thus may not be generalizable to health systems in different geographic locations. We anticipate that future studies may include manual reviews of medical records to provide additional clinical information.

## Conclusions

This is the largest study to-date to examine population level predictors of MAKE attributed to FSGS. Substantial loss of kidney function or kidney failure occurred in more than four in ten patients with FSGS and a mean baseline eGFR of 60 mL/min/1.73 m^2^ by a median of three years. We uncovered both clinical and unique non-traditional predictors of MAKE in a diverse, adult population from contemporary clinical practices of two large, geographically distinct US health systems. MAKE was predicted by lower baseline kidney function, non-corticosteroid immunomodulators, younger age, and unique features of non-commercial health insurance, more frequent outpatient visits and hospitalization. Although, lack of histopathology data precluded examination of MAKE in specific FSGS subtypes, population level predictors can improve facilitation of risk for MAKE in may benefit from testing and interventions to improve clinical outcomes.

## Electronic supplementary material

Below is the link to the electronic supplementary material.

Supplementary Material 1

## Data Availability

The data that support the findings of this study are available on request from the corresponding author. The data are not publicly available due to privacy or ethical restrictions and would require a data use agreement.

## Abbreviations

FSGS: Focal segmental glomerulosclerosis
US: United States
MAKE: Major adverse kidney events
ACE: Angiotensin converting enzyme
ARB: Angiotensin receptor blockers
eGFR: Estimated glomerular filtration rate
UCLA: University of California, Los Angeles Health
CURE-CKD: Center for Kidney Disease Research, Education, and Hope
EHR: Electronic health records for patients with
CKD: Chronic kidney disease
ICD: International Classification of Diseases
SD: Standard deviation
IQR: Interquartile range
UACR: Urine albumin/creatinine ratio
UPCR: Urine protein/creatinine ratio
CI: Confidence interval
HR: Hazard ratio
