# Supplementary material for: Population risk predictors of major adverse kidney events attributed to focal segmental glomerulosclerosis from the CURE-CKD registry
Source: BMC Nephrol. 2025 Jul 19;26:403. doi: 10.1186/s12882-025-04334-6 (PMC12276672; doi:10.1186/s12882-025-04334-6)
Supplement: Supplementary file 1 — Supplementary Material 1 [file 12882_2025_4334_MOESM1_ESM.docx]

**Additional Material**

**Table of Contents**

Additional file 1……………………………………………………………………………………………..1

Additional file 2……………………………………………………………………………………………..2

Additional file 3……………………………………………………………………………………………..3

Additional file 4……………………………………………………………………………………………..5

Additional file 5……………………………………………………………………………………………..6

| **Additional file 1.** ICD Codes for Focal Segmental Glomerulosclerosis and Kidney Failure with or without Kidney Replacement Therapy | | |
| --- | --- | --- |
| **ICD Code** | **System** | **Description** |
| **Focal Segmental Glomerulosclerosis** | | |
| N00.1 | ICD10 | Acute nephritic syndrome with focal and segmental glomerular lesions |
| N01.1 | ICD10 | Rapidly progressive nephritic syndrome with focal and segmental glomerular lesions |
| N02.1 | ICD10 | Recurrent and persistent hematuria with focal and segmental glomerular lesions |
| N03.1 | ICD10 | Chronic nephritic syndrome with focal and segmental glomerular lesions |
| N04.1 | ICD10 | Nephritic syndrome with focal and segmental glomerular lesions |
| N05.1 | ICD10 | Unspecified nephritic syndrome with focal and segmental glomerular lesions |
| N06.1 | ICD10 | Isolated proteinuria with focal and segmental glomerular lesions |
| N07.1 | ICD10 | Hereditary nephropathy, not elsewhere classified with focal and segmental glomerular lesions |
| **Kidney Failure without Kidney Replacement Therapy** | | |
| N18.5 | ICD10 | Chronic kidney disease, excludes chronic kidney disease, stage 5 requiring chronic dialysis |
| N19 | ICD10 | Unspecified kidney failure |
| **Kidney Transplant** | | |
| *Procedure* | | |
| 55.69 | ICD9-CM | Other kidney transplantation |
| 0TY00Z0 | ICD10 | Transplantation of Right Kidney, Allogenic, Open Approach |
| 0TY00Z1 | ICD10 | Transplantation of Right Kidney, Syngeneic, Open Approach |
| 0TY10Z0 | ICD10 | Transplantation of Left Kidney, Allogenic, Open Approach |
| 0TY10Z1 | ICD10 | Transplantation of Left Kidney, Syngeneic, Open Approach |
| BT29ZZZ | ICD10 | CT Scan of Kidney Transplant |
| BT49ZZZ | ICD10 | Ultrasonography of Kidney Transplant |
| *Diagnosis* | | |
| T861 | ICD10 | Complications of kidney transplant |
| T8610 | ICD10 | Unspecified complication of kidney transplant |
| T8611 | ICD10 | Kidney transplant rejection |
| T8612 | ICD10 | Kidney transplant failure |
| T8613 | ICD10 | Kidney transplant infection |
| T9619 | ICD10 | Other complication of kidney transplant |
| Z4822 | ICD10 | Encounter for aftercare following kidney transplant |
| Z940 | ICD10 | Kidney transplant status |
| **Kidney Dialysis** | | |
| *Procedure* | | |
| 39.95 | ICD9-CM | Hemodialysis |
| 54.98 | ICD9-CM | Peritoneal dialysis |
| *Diagnosis* | | |
| Z99.2 | ICD10 | Dependence on renal dialysis |
| N18.6 | ICD10 | End stage renal disease |
| ICD-international classification of diseases; CM-clinical modification | | |

| **Additional file 2**. Cox proportional hazards model summaries of MAKE survival in FSGS | | | | | | | | |
| --- | --- | --- | --- | --- | --- | --- | --- | --- |
| **Main Analysis (N=629)** | | | | | | | | |
|  | **No. outcome/No. in group** | | **Unadjusted** | | | **Adjusted** | | |
| **Variable** | **Comparison** | **Reference** | **HR** | **95% CI** | **P** | **HR** | **95% CI** | **P** |
| Age (per - 10 years) | - | - | 0.86 | 0.80-0.92 | <0.001 | 1.12 | 1.02-1.22 | 0.01 |
| Men (reference: women) | 140/342 | 122/287 | 0.97 | 0.76-1.23 | 0.79 | 0.97 | 0.75-1.24 | 0.79 |
| Non-White (reference: White) | 113/302 | 149/327 | 0.85 | 0.66-1.08 | 0.18 | 0.79 | 0.60-1.03 | 0.08 |
| Providence vs UCLA Health | 177/425 | 85/204 | 1.10 | 0.85-1.42 | 0.49 | 0.98 | 0.73-1.33 | 0.91 |
| Noncommercial vs commercial insurance | 135/258 | 127/371 | 2.02 | 1.58-2.57 | <0.001 | 1.78 | 1.36-2.33 | <0.001 |
| eGFR (per - 10 mL/min/1.73 m²) | - | - | 1.25 | 1.19-1.31 | <0.001 | 1.25 | 1.18-1.32 | <0.001 |
| Diabetes (yes/no) | 116/245 | 146/384 | 1.44 | 1.13-1.84 | 0.004 | 1.15 | 0.87-1.52 | 0.34 |
| Hypertension (yes/no) | 233/535 | 29/94 | 1.63 | 1.11-2.40 | 0.01 | 1.13 | 0.75-1.71 | 0.56 |
| ACE inhibitor/ARB (yes/no) | 217/475 | 45/154 | 1.53 | 1.11-2.10 | 0.01 | 1.22 | 0.87-1.71 | 0.25 |
| Corticosteroid (yes/no) | 130/297 | 132/332 | 1.16 | 0.91-1.48 | 0.24 | 0.77 | 0.59-1.02 | 0.07 |
| Other immunomodulator^1^ (yes/no) | 43/74 | 219/555 | 1.76 | 1.27-2.44 | 0.001 | 1.87 | 1.32-2.65 | <0.001 |
| Outpatient Visits (average per quarter) | - | - | 1.03 | 1.02-1.05 | <0.001 | 1.03 | 1.01-1.05 | 0.001 |
| Hospitalization (yes/no) | 95/181 | 167/448 | 2.04 | 1.58-2.63 | <0.001 | 1.64 | 1.25-2.15 | <0.001 |
| **Exploratory analysis of patients with UACR/UPCR measurements (N=299)** | | | | | | | | |
|  | **No. outcome/No. in group** | | **Unadjusted** | | | **Adjusted** | | |
| **Variable** | **Comparison** | **Reference** | **HR** | **95% CI** | **P** | **HR** | **95% CI** | **P** |
| Age (per - 10 years) | - | - | 0.89 | 0.80-0.98 | 0.02 | 1.08 | 0.94-1.25 | 0.27 |
| Men (reference: women) | 69/165 | 56/134 | 1.04 | 0.73-1.48 | 0.81 | 1.12 | 0.77-1.63 | 0.56 |
| Non-White (reference: White) | 60/157 | 65/142 | 0.94 | 0.66-1.34 | 0.73 | 0.85 | 0.57-1.25 | 0.41 |
| Providence vs UCLA Health | 72/188 | 53/111 | 0.76 | 0.53-1.08 | 0.12 | 0.62 | 0.40-0.96 | 0.03 |
| Noncommercial vs commercial insurance | 61/111 | 64/188 | 1.97 | 1.39-2.81 | <0.001 | 1.72 | 1.13-2.62 | 0.01 |
| eGFR (per - 10 mL/min/1.73 m²) | - | - | 1.23 | 1.15-1.32 | <0.001 | 1.23 | 1.13-1.34 | <0.001 |
| UACR >1430mg/g UPCR >1.6g/g (yes/no) | 91/156 | 34/143 | 3.78 | 2.54-5.63 | <0.001 | 3.46 | 2.28-5.23 | <0.001 |
| Diabetes (yes/no) | 67/140 | 58/159 | 1.38 | 0.97-1.97 | 0.07 | 1.03 | 0.67-1.59 | 0.90 |
| Hypertension (yes/no) | 114/264 | 11/35 | 1.61 | 0.86-2.99 | 0.13 | 0.74 | 0.35-1.54 | 0.42 |
| ACE inhibitor/ARB (yes/no) | 112/237 | 13/62 | 2.61 | 1.47-4.64 | 0.001 | 2.17 | 1.20-3.95 | 0.01 |
| Corticosteroid (yes/no) | 61/139 | 64/160 | 1.15 | 0.81-1.63 | 0.43 | 0.81 | 0.53-1.24 | 0.34 |
| Other immunomodulator^1^ (yes/no) | 20/29 | 105/270 | 2.50 | 1.55-4.04 | <0.001 | 2.93 | 1.76-4.86 | <0.001 |
| Outpatient Visits (average per quarter) | - | - | 1.04 | 1.02-1.06 | <0.001 | 1.02 | 1.00-1.05 | 0.10 |
| Hospitalization (yes/no) | 41/73 | 84/226 | 2.12 | 1.46-3.09 | <0.001 | 1.91 | 1.22-2.99 | 0.005 |
| MAKE-major adverse kidney events, FSGS-focal segmental glomerulosclerosis, HR-hazard ratio, CI-confidence interval, UCLA-University of California, Los Angeles, eGFR-estimated glomerular filtration rate, ACE-angiotensin converting enzyme, ARB-angiotensin II receptor blocker, UACR-urine albumin/creatinine ratio, UPCR-urine protein/creatinine ratio.  ^1^includes biologics, calcineurin inhibitors, cytotoxic agents, mammalian target of rapamycin inhibitors, corticotropin agents, and pyrimidine synthesis inhibitors. | | | | | | | | |

**Additional file 3**. Characteristics of patients identified with FSGS by eGFR category at baseline

|  |  | Baseline eGFR, mL/min/1.73 m^2^ | | | | |
| --- | --- | --- | --- | --- | --- | --- |
|  | **Overall** | **≥90** | **60–89** | **45–59** | **30–44** | **15–29** |
| Men, n (%) | 342 (54) | 67 (52) | 84 (55) | 59 (57) | 83 (59) | 49 (47) |
| Care at Providence, n (%) | 425 (68) | 83 (65) | 91 (59) | 77 (74) | 96 (69) | 78 (75) |
| Non-White, n (%) | 302 (48) | 70 (55) | 71 (46) | 51 (49) | 57 (41) | 53 (51) |
| Hypertension, n (%) | 535 (85) | 97 (76) | 125 (82) | 93 (89) | 127 (91) | 93 (89) |
| Diabetes, n (%) | 245 (39) | 44 (34) | 60 (39) | 36 (35) | 64 (46) | 41 (39) |
| Hospitalization, n (%) | 181 (29) | 28 (22) | 37 (24) | 30 (29) | 45 (32) | 41 (39) |
| Non-corticosteroid immunomodulator, n (%) | 74 (12) | 15 (12) | 22 (14) | 14 (13) | 15 (11) | 8 (8) |
| Non-commercial insurance, n (%) | 258 (41) | 38 (30) | 52 (34) | 44 (42) | 69 (49) | 55 (53) |
| Corticosteroid, n (%) | 297 (47) | 57 (45) | 74 (48) | 51 (49) | 68 (49) | 47 (45) |
| ACE inhibitor/ARB, n (%) | 475 (76) | 83 (65) | 117 (76) | 77 (74) | 115 (82) | 83 (80) |
| Age (decades), mean, SD | 5, 2 | 4, 1 | 5, 2 | 5, 2 | 6, 2 | 6, 2 |
| eGFR, mean, SD | 6.0, 3.0 | 106, 12 | 73, 9 | 51, 5 | 37, 4 | 22, 4 |
| Outpatient visits, mean, SD | 7, 8 | 6, 5 | 8, 8 | 7, 10 | 7, 7 | 5, 6 |

FSGS-focal and segmental glomerulosclerosis, eGFR-estimated glomerular filtration rate, ACE-angiotensin converting enzyme, ARB-angiotensin II receptor blocker

**Additional file 4.** Summary of unadjusted Cox P = proportional hazards models by eGFR category

**
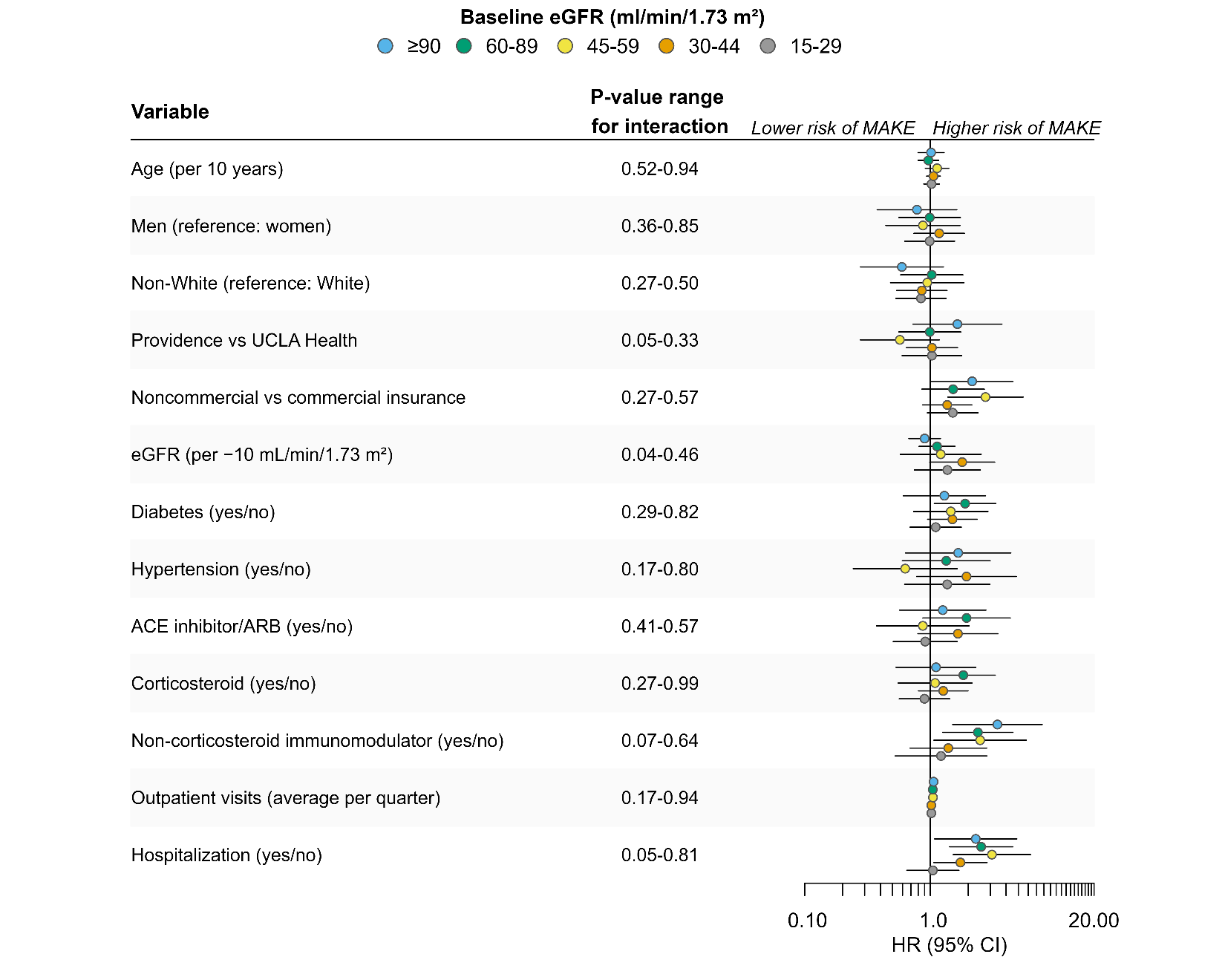
**

eGFR-estimated glomerular filtration rate, ACE-angiotensin converting enzyme, ARB-angiotensin II receptor blocker,

**Additional file 5.** Summary of outcomes among patients with FSGS and MAKE by eGFR category

|  |  | Baseline eGFR, mL/min/1.73 m^2^ | | | | |
| --- | --- | --- | --- | --- | --- | --- |
|  | **Overall** | **≥90** | **60–89** | **45–59** | **30–44** | **15–29** |
| First event, n (%) | 262 (41.7) | 29 (22.7) | 49 (32.0) | 34 (32.7) | 76 (54.3) | 74 (71.2) |
| eGFR 40% decline, n (% of events) | 133 (50.8) | 22 (75.9) | 40 (81.6) | 25 (73.5) | 41 (53.9) | 5 (6.8) |
| Kidney failure | 74 (28.2) | 3 (10.3) | 2 (4.1) | 4 (11.8) | 17 (22.4) | 48 (64.9) |
| Dialysis | 28 (10.7) | 1 (3.4) | 2 (4.1) | 2 (5.9) | 8 (10.5) | 15 (20.3) |
| Transplant | 13 (5.0) | 2 (6.9) | 4 (8.2) | 2 (5.9) | 2 (2.6) | 3 (4.1) |
| All-cause death | 14 (5.3) | 1 (3.4) | 1 (2.0) | 1 (2.9) | 8 (10.5) |  |

FSGS-focal and segmental glomerulosclerosis, MAKE-major adverse kidney events, eGFR-estimated glomerular filtration rate
